# Self-Healing Property of Asphalt Mixtures Containing Corn Oil Microcapsules

**DOI:** 10.3390/ma19112216

**Published:** 2026-05-25

**Authors:** Yuejing Lv, Jinlin Cheng

**Affiliations:** 1 Department of Traffic Engineering, School of Automobile and Traffic Engineering, Wuhan University of Science and Technology, Wuhan 430065, China; chengjinlinmobile@163.com; 2 Qinghai Provincial Highway Bureau, Xining 810001, China

**Keywords:** asphalt, corn oil microcapsules, in situ polymerization, self-healing property, splitting test, microcapsule content

## Abstract

Asphalt pavements are prone to the formation of microcracks due to aging under environmental factors, and microcapsule-based self-healing technology represents an effective means of preventive maintenance. In this study, corn oil, a renewable and environmentally friendly material, was selected as the asphalt rejuvenator to prepare corn oil microcapsules via in situ polymerization, and the self-healing performance of corn oil microcapsule-modified asphalt was investigated. By analyzing the effects of corn oil microcapsules on the high-temperature performance, salt resistance, chemical structure, and microscopic morphology of asphalt, as well as the influence of temperature, time, and corn oil microcapsule content on the self-healing performance of asphalt mixtures, the self-healing mechanism of corn oil microcapsule-modified asphalt was elucidated at both the microscopic and macroscopic levels. The results showed that during the preparation of corn oil microcapsules, the optimal molar ratio of M_F_:M_(M+U)_ was 2.5, with an emulsification rate of 1.2 kr/min. The prepared corn oil microcapsules exhibited high yield and good encapsulation efficiency, possessed excellent high-temperature resistance that met the requirements of the asphalt mixing stage, and showed superior salt resistance. FTIR analysis confirmed the successful incorporation of microcapsules into the asphalt system. Atomic force microscopy (AFM) observations revealed that the microcapsules mitigated microscopic surface damage caused by aging. The healing index of the asphalt mixtures incorporating corn oil microcapsules increased with prolonged healing time and elevated temperature. By establishing the relationship between the healing index and the content of corn oil microcapsules, the recommended content of corn oil microcapsules within the tested range is 6 wt%. This study elucidates the self-healing mechanism of corn oil microcapsule-modified asphalt from both microscopic (surface parameter recovery) and macroscopic (mechanical property restoration) scales, providing a scientific basis for the application of microcapsule technology in green and sustainable asphalt pavement maintenance.

## 1. Introduction

Asphalt materials gradually age due to the cleavage of C=C bonds under the influence of factors such as wind, light, moisture, and temperature, leading to the formation of microcracks in asphalt pavements. Unaddressed microcracks in asphalt pavements can further develop into issues such as pavement cracks and potholes [1].

Asphalt self-healing technology is an important means of controlling the further development of microcracks and maintaining the good working condition of asphalt pavements. Currently, self-healing in asphalt pavements can be achieved through the incorporation of modifiers [2,3], induction heating [4], microcapsules [5], and other techniques. However, existing asphalt self-healing technologies require controlling numerous additional conditions. For instance, while incorporating modifiers can enhance the initial performance of asphalt, it presents issues such as high cost and performance degradation over long-term service due to the aging of the modifiers themselves. Furthermore, techniques like induction heating can raise the temperature of the asphalt pavement, generate harmful gases, and accelerate the aging process of the asphalt, which limits their widespread adoption. As an emerging, low-carbon, and practical technology, microcapsules have been widely regarded in recent years as an effective approach to enhance the self-healing capacity of asphalt materials. By selecting suitable asphalt rejuvenators as core materials and encapsulating them with shell materials to form a microcapsule structure, microcapsules can be incorporated into asphalt mixtures and uniformly distributed within the pavement structure. During the service life of the asphalt pavement with embedded microcapsules, asphalt aging leads to stress concentration, causing the microcapsules in critical areas to undergo increased stress, crack, and rupture [6]. The released rejuvenator from the microcapsules then diffuses into the cracks, reconstructing the chemical composition of the aged asphalt and restoring its performance. Therefore, developing this technology can repair cracks at their initial stage, helping to reduce maintenance costs and achieve energy conservation and emission reduction.

The preparation processes of asphalt microcapsules vary, including adsorption encapsulation, interfacial polymerization, and in situ polymerization. Adsorption encapsulation operates through physical processes, where the core material is emulsified, atomized, and shaped by solvent evaporation. While this method is simple to operate, the resulting microcapsules are millimeter-scale particles with relatively large particle sizes. When incorporated into asphalt mixtures, these oversized microcapsules can, to a certain extent, adversely affect the fundamental skeleton structure of the mixture [7]. Interfacial polymerization utilizes two reactive monomers that rapidly undergo polycondensation at the oil-water interface to form a membrane. The microcapsules produced by this method exhibit high encapsulation efficiency, fast reaction rates, and a dense shell. However, it often leaves behind residual unreacted monomers that are difficult to completely remove [8]. In contrast, in situ polymerization involves the pre-polymerization of wall-forming monomers in the continuous phase, followed by the deposition of the prepolymer onto the surface of emulsified core droplets under controlled pH and temperature conditions, where it crosslinks to form a shell. Microcapsules prepared by this method offer low cost and high encapsulation efficiency, allow for control over the shell thickness, and are advantageous for industrial production due to their cost-effectiveness [9]. Therefore, the optimal preparation process for microcapsules via in situ polymerization was determined.

Mohanty et al. [10] utilized vegetable oil rejuvenators to regenerate aged asphalt and found that vegetable oil could effectively mitigate the adverse effects of aging on asphalt viscosity and low-temperature performance. Peng et al. [11] conducted performance tests on various vegetable oil rejuvenators and observed that corn oil exhibited superior performance in certain specific indicators. Compared with traditional mineral-based rejuvenators, corn oil exhibits the characteristics of moderate viscosity, low volatility, and good compatibility with aged asphalt, enabling it to effectively replenish the light components lost during aging and regulate the chemical composition balance of the asphalt. As a vegetable oil derived from renewable resources, corn oil is characterized by its wide availability, low cost, and environmental friendliness, with its main components being unsaturated fatty acids such as oleic acid and linoleic acid, making it an ideal candidate material for preparing asphalt rejuvenators. Furthermore, the application of corn oil aligns with the principles of green highway construction and sustainable development.

Therefore, this study proposes the use of corn oil as an asphalt rejuvenator to prepare microcapsules via in situ polymerization. High-temperature resistance, salt resistance, and FTIR tests were conducted on corn oil microcapsules. The microscopic surface characteristics of microcapsule asphalt were quantitatively analyzed using atomic force microscopy, and the macroscopic splitting test of asphalt mixtures was employed to evaluate the self-healing performance of corn oil microcapsules. In this study, corn oil was used for the first time as the core material of self-healing microcapsules for asphalt. This work not only expands the alternative pool of asphalt rejuvenators but also consolidates the theoretical foundation of self-healing microcapsule technology, thereby providing theoretical support for the recycling and reclamation of asphalt pavement materials.

## 2. Materials and Methods

### 2.1. Materials

#### 2.1.1. Corn Oil Microcapsules

(1) Raw Material Ratio

Based on the different mass ratios of raw materials listed in Table 1, preliminary experiments for the preparation of asphalt microcapsules were designed in this study. The urea mass ratio refers to the proportion of urea mass relative to the prepolymer mass; M_F_: M_(M+U)_ denotes the molar ratio of formaldehyde to the sum of melamine and urea. By controlling the ratios of melamine, urea, and formaldehyde to create comparative groups, the optimal formulation was determined [12].

**Figure 1:**
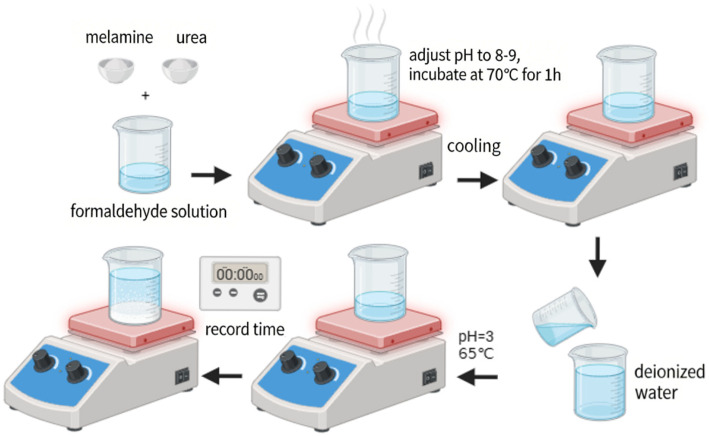
Flowchart of the prepolymer synthesis experiment.

The experimental procedure for prepolymer synthesis is shown in Figure 1, with the main steps as follows:

(1) A total of 5 g of melamine, urea, and formaldehyde was added to 25 mL of deionized water. The mixture was stirred until the urea was completely dissolved, and the pH of the mixed solution was adjusted to 8–9 using a standard sodium hydroxide solution. Then, under stirring conditions, the temperature of the mixed solution was slowly raised from room temperature to 70 °C and maintained at this temperature for 1 h. Afterward, the reaction was terminated, and the prepolymer was slowly cooled to room temperature.
(2) The prepolymer was added to 150 mL of deionized water under continuous stirring, and the pH of the mixed solution was adjusted to 3 using a dilute sulfuric acid solution. Meanwhile, the temperature of the mixed solution was increased to 65 °C at an appropriate rate. Finally, the time required for the formation of a large amount of white polymer in the reaction vessel was recorded [13].

The experimental results are shown in Table 2. Analysis indicates that when the urea mass ratio is 0%, the polymerization rate of melamine and formaldehyde is very fast under the action of an acidic catalyst. This phenomenon is unfavorable for the deposition of the prepolymer on the surface of corn oil, making it difficult to form a shell structure and thus leading to the failure of corn oil microcapsule preparation. As the urea content gradually increases from 0% to 80%, the polymer formation time increases from 14 min to 96 min. This is because when melamine is largely replaced by urea, the excessively long formation time and unstable emulsion negatively affect the demulsification efficiency. Repeated experimental results show that when the urea mass ratio is 20%, the polymer formation time is appropriate, and the microcapsule product yield is high. Therefore, the formula for the shell material is fixed as No. 3, meaning that urea accounts for 20% of the prepolymer mass.

(2) Emulsification Rate

**Figure 2:**
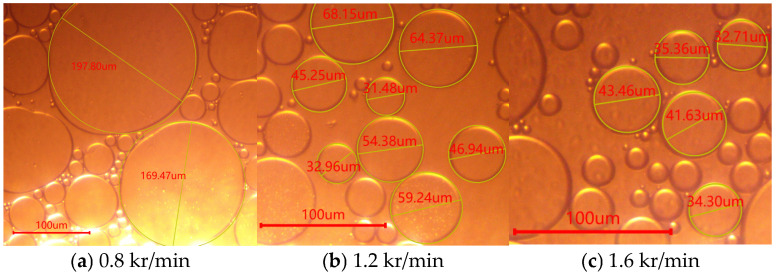
Fluorescence microscopy (FM) images of the emulsion at different stirring rates.

When preparing microcapsules via in situ polymerization, their size is primarily determined by the droplet size formed during the emulsification of the rejuvenator. During synthesis, an appropriate emulsification rate significantly impacts the quality of the microcapsules [14]. To determine the optimal stirring rate for the emulsification process, preliminary emulsification experiments were conducted in this study, with the main steps as follows:

(1) 0.7 g of sodium dodecylbenzenesulfonate powder was added to 100 mL of an aqueous solution, which was then placed under a high-speed shear emulsifier and stirred uniformly at 0.4 kr/min for 30 min. Subsequently, 5 g of corn oil was poured into the solution and emulsified at shear rates of 0.8 kr/min, 1.2 kr/min, and 1.6 kr/min for 1 h each, resulting in emulsified solutions.
(2) Approximately 2 µm of the emulsified solution was drawn using a microsyringe, dropped onto a glass slide, and observed under a fluorescence microscope in bright field. The observation results are shown in Figure 2.

Analysis of Figure 2 reveals that as the shear rate increases, the average particle size of corn oil droplets decreases, and the distribution range gradually narrows. When the shear rate is 0.8 kr/min, a large number of corn oil droplets with particle sizes exceeding 100 μm are present in the solution, as shown in Figure 2, indicating that the shear rate is too low and a significant amount of corn oil is insufficiently emulsified. During the experiment, it was observed that when the emulsification rate was 1.6 kr/min, a large number of bubbles formed in the dispersion system, which hindered the second-stage synthesis of microcapsules. Considering both the quality and average size of the microcapsules, the emulsification rate was determined to be 1.2 kr/min. At this rate, the particles in the emulsion are well-formed, with uniform particle sizes, and no significant bubble formation occurs to affect subsequent experiments.

(3) Optimized Preparation Process

**Figure 3:**
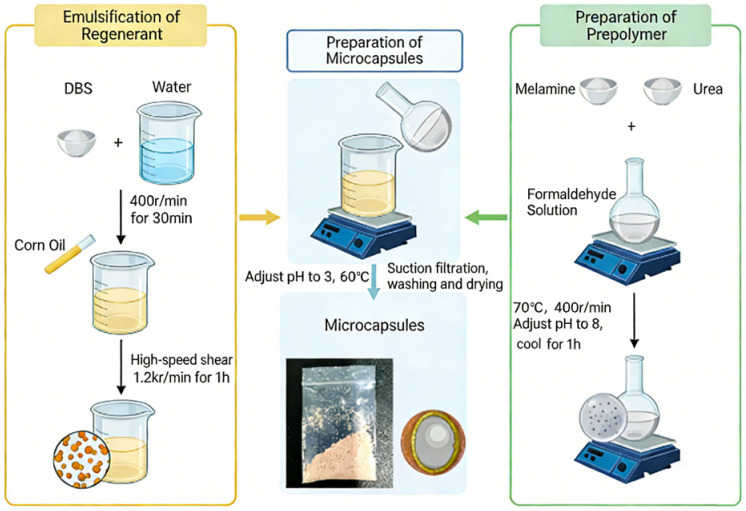
Flowchart of microcapsule preparation.

Based on the aforementioned research and preliminary experimental findings, the preparation of microcapsules was determined to follow the experimental procedure illustrated in Figure 3:

(1) Preparation of the Prepolymer

Into 25 mL of deionized water, melamine, urea, and formaldehyde were sequentially added at a weight ratio of 1:2:7. The mixture was placed in a 70 °C water bath and reacted with stirring at 0.4 kr/min until the urea was completely dissolved. Subsequently, the pH of the reaction system was adjusted to 8.0 using an alkaline solution, and the reaction was continued for 1 h before termination. After allowing the reaction solution to cool to room temperature, the melamine-urea-formaldehyde prepolymer solution was obtained.

(2) Emulsification of the Rejuvenator

An appropriate amount of sodium dodecylbenzenesulfonate (DBS) was weighed, dissolved in 100 mL of deionized water, and then placed under a high-shear emulsifier. The mixture was stirred uniformly at 0.4 kr/min for 30 min to ensure complete dissolution of the DBS. Corn oil was then slowly dripped into this solution, and the shear rate was increased to 1.2 kr/min for continuous emulsification over 1 h, ultimately yielding a stable emulsified solution.

(3) Synthesis of Microcapsules

The prepolymer solution was poured into the emulsified solution, followed by the addition of an appropriate amount of curing agent and thorough mixing. Subsequently, the temperature of the mixed system was raised to 60 °C, and a dilute acid solution was used to adjust the pH to 3.0 to initiate the curing reaction. After 4 h of reaction, the system’s pH was adjusted back to 7.0 using a dilute alkaline solution to terminate the reaction. Once the reaction solution had cooled to room temperature, the resulting turbid suspension was collected. After undergoing suction filtration, washing with deionized water, and drying, the target microcapsule powder was obtained [15].

#### 2.1.2. Preparation of Self-Healing Asphalt

70# asphalt was selected as the main experimental material. Aged asphalt was obtained by aging the base asphalt in a Rolling Thin Film Oven (RTFOT) at 163 °C for 4 h. The RTFOT was manufactured by Shanghai Changji Geological Instrument Co., Ltd. (Shanghai, China). The aged asphalt was then placed in a container and heated to 100 °C for subsequent use. The primary objective was to verify the feasibility of the microcapsule-based self-healing strategy, rather than to optimize the microcapsule dosage. Therefore, we selected two representative dosages (4% and 8%) to demonstrate the improvement in self-healing ability conferred by the microcapsules. Corn oil microcapsules were added at mass fractions of 4% and 8%, respectively, and were stirred uniformly until the microcapsules were evenly distributed within the asphalt, resulting in two self-healing asphalt samples with different microcapsule dosages [16].

#### 2.1.3. Asphalt Mixture Incorporating Corn Oil Microcapsules

The asphalt used in the tests was 70# base asphalt. The coarse and fine aggregates were limestone, conforming to the requirements of the JTG E42-2005 [17].

The preparation process for the corn oil microcapsule-modified asphalt was as follows: First, the base asphalt was heated to 135 °C. A specific mass of corn oil microcapsules was then added to the molten asphalt, and the mixture was stirred continuously at 1 kr/min for 30 min to produce the corn oil microcapsule-modified asphalt [18].

**Figure 4:**
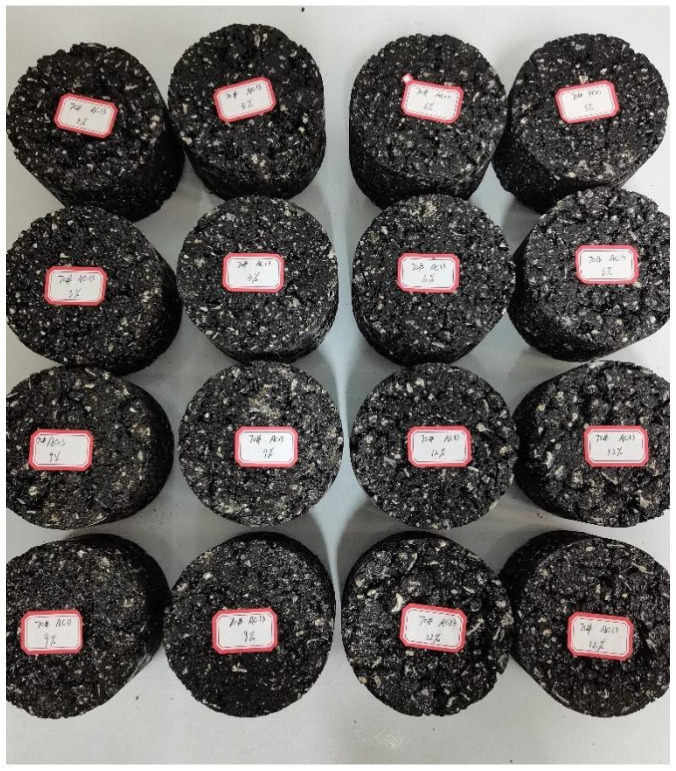
Marshall specimens of microcapsule-modified asphalt mixture.

The AC-13 asphalt mixture design was completed in this study using the Marshall test method [19,20]. The corn oil microcapsule content was determined as a percentage of its mass relative to the asphalt mass and set at 0%, 3%, 6%, 9%, and 12%, respectively. The corn oil microcapsule-modified asphalt with different dosages was mixed with aggregates and mineral filler—employing a wet process—to prepare the corn oil microcapsule-modified asphalt mixtures. Finally, Marshall specimens were compacted with an asphalt-aggregate ratio of 4.7%. Their appearance is shown in Figure 4.

### 2.2. Test Methods

#### 2.2.1. Performance Tests of Corn Oil Microcapsules

(1) Micromorphology and Particle Size Tests

**Figure 5:**
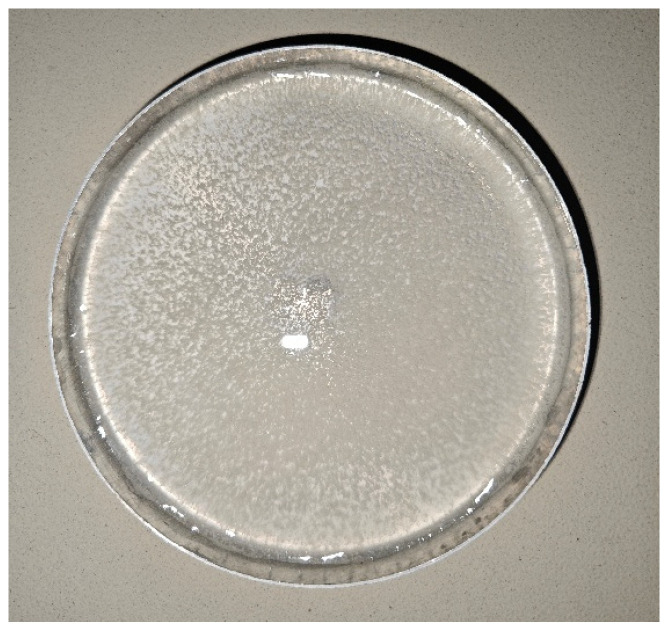
Aqueous suspension of corn oil microcapsules.

A fluorescence microscope (FM, model XSP-63X) was used to obtain the microscopic morphology of the microcapsules and examine the encapsulation of corn oil by the shell material [21]. The FM was manufactured by Jiangxi Phoenix Optical Technology Co., Ltd. (Shangrao, China). To prevent the agglomeration of the microcapsule powder, the powder was first dispersed in deionized water to form an aqueous solution, which was then stirred at low speed in a homogenizer for 30 min to ensure the uniform dispersion of the microcapsules, as shown in Figure 5. An appropriate amount of the microcapsule aqueous solution was drawn and dropped onto a glass slide for dark-field observation under the FM.

(2) Thermogravimetric Analysis (TGA) Test

In this experiment, the thermal stability of the prepared corn oil microcapsule powder was characterized using a Simultaneous Thermal Analyzer (STA). The STA was manufactured by NETZSCH (Selb, Germany). The test was performed in a nitrogen atmosphere with a heating rate of 10 °C/min.

(3) Salt Resistance Test

To evaluate the salt resistance of the corn oil microcapsules, this study immersed the microcapsules in NaCl solutions with mass fractions of 0%, 5%, and 10% for treatment. The specific procedure was as follows: 2 g of the microcapsule sample was weighed and continuously treated in a water bath at 50 °C with stirring at 0.65 kr/min for 120 min, followed by standing at room temperature for 24 h. To determine the retention of the core material after exposure to the salt solutions, the treated samples were analyzed using a petroleum ether extraction method. Unlike conventional grinding treatment, this experiment first dried the samples and then directly soaked them in petroleum ether to extract the corn oil that may have been released due to the salt solution treatment. The extraction rate Y of corn oil was calculated using Formula (1) to assess the salt resistance of the corn oil microcapsules [22].

(1) $$Y=\frac{m_{1}-m_{2}}{m_{1}}\times 100\%$$

where Y is the extraction content/%; $m_{1}$ is the mass of the corn oil microcapsule sample/g; $m_{2}$ is the mass of the filter residue/g.

(4) Fourier Transform Infrared Spectroscopy (FTIR) Test

To systematically analyze the influence of corn oil microcapsules on the chemical structure of asphalt, Fourier transform infrared spectroscopy (FTIR) tests were conducted on asphalt samples containing 0%, 2%, 4%, and 6% corn oil microcapsules, with 0.1 g taken from each sample. The FTIR was manufactured by Bruker (Santa Barbara, CA, USA). To verify the uniformity of microcapsule dispersion within the samples and the reliability of the experimental results, samples for each dosage were taken from three different locations, and the FTIR test was repeated three times [23]. The results showed that the spectra from the three tests for each dosage group were highly consistent, indicating that the corn oil microcapsules were uniformly distributed within the asphalt matrix with good compatibility and dispersion stability, thereby ensuring the accuracy of the subsequent structural analysis [24].

#### 2.2.2. Surface Microstructure Test

The local microstructural characteristics of the self-healing asphalt samples were scanned and analyzed using a Dimension ICON atomic force microscope (AFM). The AFM was manufactured by Bruker (Santa Barbara, CA, USA).

#### 2.2.3. Splitting Tensile Test

To evaluate the influence of corn oil microcapsules on the healing performance of asphalt mixtures, a process simulating the damage–healing–redamage of corn oil microcapsule-modified asphalt mixtures was designed. Using a multi-function automatic asphalt pressure tester (SYD-0730A), a two-stage splitting test was conducted on each Marshall specimen of corn oil microcapsule-modified asphalt [25]. The multi-function automatic asphalt pressure tester was manufactured by Shanghai Changji Geological Instrument Co., Ltd. (Shanghai, China). Different curing conditions were applied between the two splitting tests to assess the effects of various factors, such as healing time, healing temperature, and corn oil microcapsule content, on the self-healing performance of the asphalt mixture.

**Figure 6:**
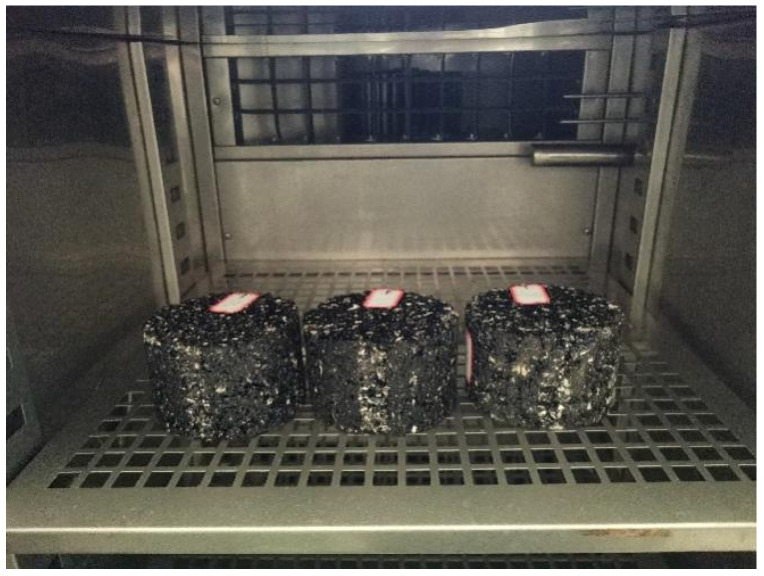
Curing environment inside the constant temperature chamber.

The method for testing healing performance is primarily implemented through the following steps:

(1) First, the specimen is placed at 15 °C for at least 2 h for conditioning. The Marshall specimen is then removed, and a labeled arrow is attached to its side to ensure the consistency in the loading application points between the two-stage splitting tests [26]. Subsequently, a loading rate of 2 mm/min was set, and the specimen was loaded to create microcracks. After loading is complete, the specimen is removed from the instrument and set aside for later use. The maximum load $P_{T1}$ at this stage is recorded in units of kN.

(2) After the first-stage splitting test, the damaged Marshall specimen is obtained. It is bound with two rubber bands perpendicular to the splitting direction, then transferred into an environmental chamber for healing under specified time and temperature conditions, as shown in Figure 6.

(3) After curing is completed, the Marshall specimen is stored at 15 °C for 2 h. The rubber bands are then removed, and the procedure in step (1) is repeated, with the difference being that the loading rate is set to 50 mm/min in order to completely split the Marshall specimen [27]. The maximum load $P_{T2}$ at this stage is recorded in units of kN. The ratio of $P_{T2}$ to $P_{T1}$ is defined as the healing index, calculated as follows:

(2) $$\mathrm{HI}=\frac{1}{3}\left(\sum_{i=1}^{3}\frac{P_{T2}}{P_{T1}}\right)$$

where  HI is the healing index of the Marshall specimen (dimensionless); i is the number of parallel tests;$\;P_{T1}$ is the maximum load during the first-stage splitting test in the healing experiment;${\;P}_{T2}$ is the maximum load during the second-stage splitting test in the healing experiment.

## 3. Results and Discussion

### 3.1. Basic Performances of Corn Oil Microcapsules

#### 3.1.1. Morphological Characteristics and Particle Size Distribution

**Figure 7:**
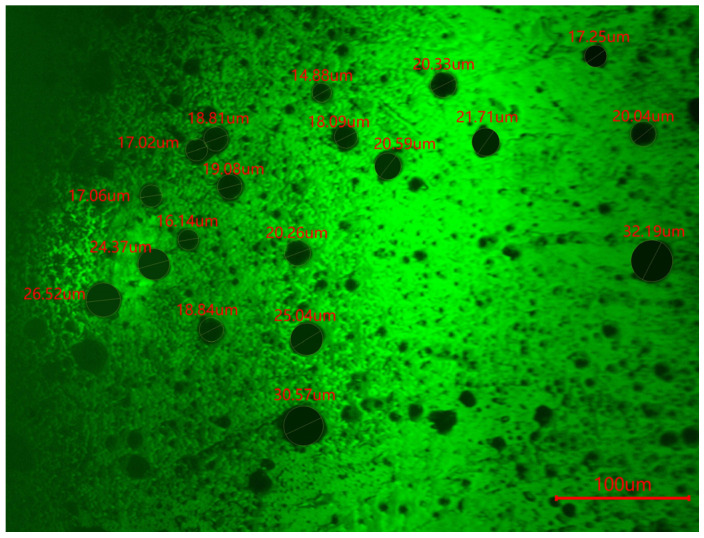
Microscopic morphology of corn oil microcapsules under FM.

As shown in Figure 7, the dark-green irregular spherical particles observed were the corn oil microcapsules.

Using ImageView (version 3.7.10121.20171030), an area containing at least 50 microcapsule particles in the FM image was selected for microcapsule counting and particle size analysis. A total of 60 structurally intact microcapsule particles were observed. The particle size of the corn oil microcapsules mainly ranged from 0 to 30 μm, accounting for 75% of the total, with an average particle size of 23.7 μm, which falls within the micrometer-scale microcapsule range. The size of the microcapsules is related to the emulsification process; the more stable the emulsion system, the more uniform the microcapsule particles. The experimental results indicate that the emulsion system formed by corn oil and DBS was effective, producing uniform oil droplets after emulsification. The polymer stably adhered to the surface of the oil droplets after mixing with the emulsion, forming the corn oil microcapsules.

#### 3.1.2. Analysis of High-Temperature Resistance

**Figure 8:**
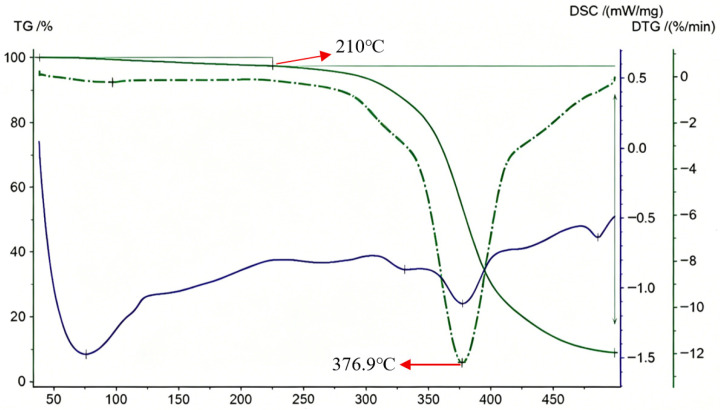
TGA test results of corn oil microcapsules.

As shown in Figure 8, within the temperature range of 0 °C to 210 °C, the sample mass remained essentially stable, ensuring the structural integrity of the corn oil microcapsules during the asphalt mixing stage [28]. Before 210 °C, the decomposition rate of the corn oil microcapsule sample was extremely low, indicating that the temperature increase did not cause significant mass loss. Subsequently, the decomposition rate gradually increased, reaching a maximum decomposition rate of 12.4%/min at 376.9 °C. This phenomenon indicates that significant thermal decomposition of the corn oil microcapsules occurred within the range of 210 °C to 376.9 °C. Considering that the maximum temperature likely to be encountered during the preparation and practical road application of corn oil microcapsules is typically below 200 °C, it can be concluded that these corn oil microcapsules possess thermal stability that fundamentally meets the usage requirements.

#### 3.1.3. Analysis of Salt Resistance

The experimental results are shown in Table 3. When the corn oil microcapsules were immersed in an aqueous solution, the extraction rate of the core material was only 3%. As the concentration of the NaCl solution increased from 0 wt% to 10 wt%, the extracted amount of corn oil gradually rose from 3% to 19.5%. This is mainly because a portion of the microcapsules ruptured in the high-concentration salt solution, leading to the release of the core material. In practical engineering applications of asphalt pavements, sodium chloride (NaCl) is widely used as a deicing salt for pavement snow melting, with a commonly adopted concentration of approximately 10%. After immersion in the 10% NaCl solution, the leaching rate of corn oil from the microcapsules was still only 19.5%, which indicates that the prepared microcapsules possess favorable salt resistance.

#### 3.1.4. Spectral Characteristics of Corn Oil Microcapsule-Modified Asphalt

**Figure 9:**
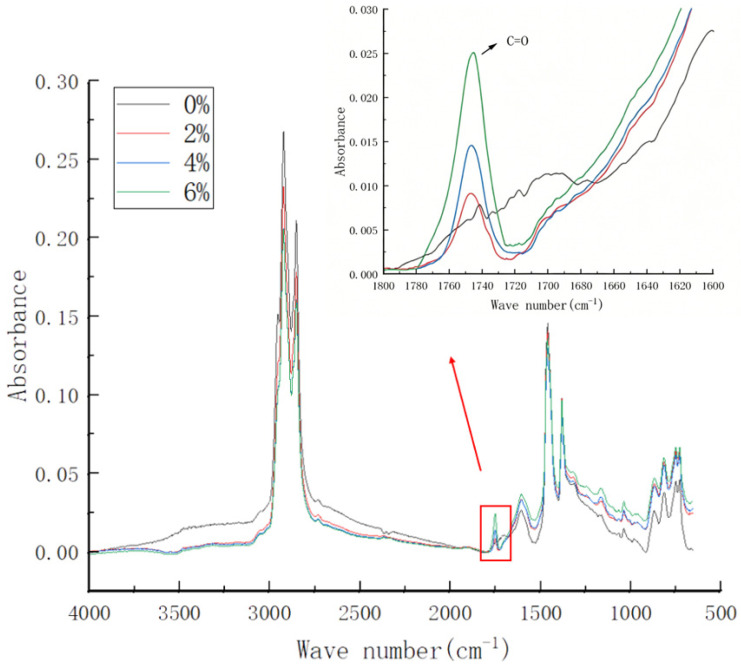
FTIR spectra of asphalt mixtures with different corn oil microcapsule contents.

The comparative results of the FTIR spectra for asphalt with different dosages of corn oil microcapsules are shown in Figure 9. Here, 70-0 represents the base 70# asphalt, 70-2 represents the 70# asphalt sample with 2% corn oil microcapsules, and so on. As seen in Figure 9, compared to the 70-0 sample without microcapsules, the asphalt samples containing corn oil microcapsules exhibited a distinct absorption peak near 1747 cm^−1^. This peak corresponds to the stretching vibration of the C=O bond in saturated fatty acid esters, which is a characteristic absorption peak introduced by the corn oil microcapsules. As the microcapsule dosage gradually increased, the intensity of this characteristic peak showed a regular enhancement, indicating a corresponding increase in the relative content of C=O functional groups. This absorption peak can be regarded as a characteristic feature resulting from the addition of corn oil microcapsules.

### 3.2. Microscale Properties of Self-Healing Asphalt

#### 3.2.1. Analysis of Apparent Morphological Characteristics of Self-Healing Asphalt

**Figure 10:**
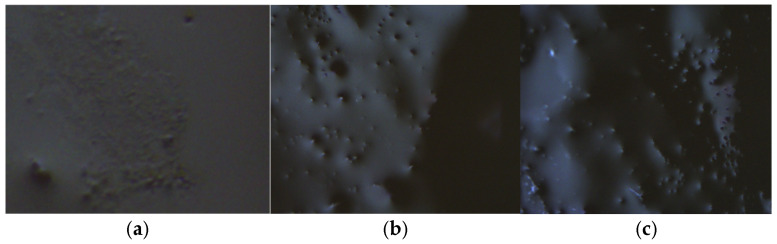
Distribution of corn oil microcapsules in self-healing asphalt at different contents. (**a**) Aged asphalt; (**b**) Aged asphalt with 4% microcapsules; (**c**) Aged asphalt with 8% microcapsules.

The distribution of corn oil microcapsules in asphalt is shown in Figure 10. Analysis shows that the microcapsules are uniformly dispersed in asphalt at different contents, and their number on the asphalt surface increases with increasing microcapsule content. The microcapsules remain intact and show no severe agglomeration within the tested content range, demonstrating excellent interfacial compatibility with asphalt. This favorable dispersion characteristic is crucial for achieving consistent and reliable self-healing effects throughout the asphalt material.

#### 3.2.2. Microstructure Analysis of Self-Healing Asphalt

**Figure 11:**
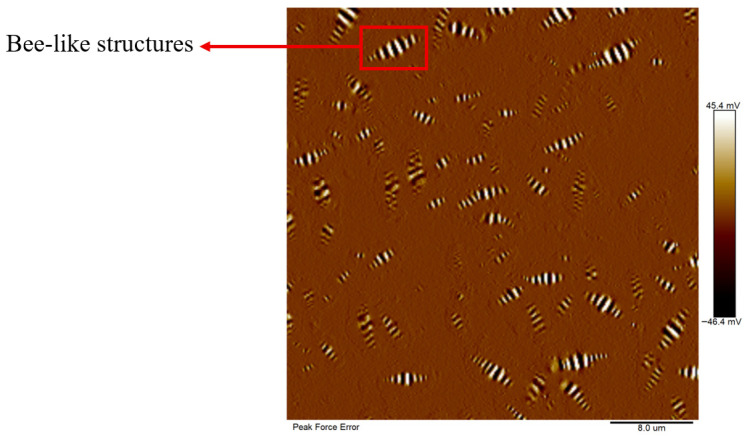
Bee-like structures of self-healing asphalt with corn oil microcapsules.

Four types of samples (base asphalt, aged asphalt, and aged asphalt containing 4% and 8% corn oil microcapsules) were characterized by tapping-mode atomic force microscopy (AFM). As shown in Figure 11, AFM observations revealed distinct alternating strip-like morphologies on the asphalt surface, commonly known as “bee-like structures” in the literature [29]. These features arise from alternating protrusions oriented in two perpendicular directions, where bright domains correspond to elevated wax-rich peaks and dark domains correspond to depressed asphalt-rich valleys. The peak regions are predominantly composed of crystalline wax, with asphaltenes and resins adsorbed onto their surfaces [30].

(1) Analysis of Bee-like Structures

To quantitatively analyze the influence of microcapsule content on the bee-like structures of the asphalt surface, software such as Image-Pro Plus (version 10.0.14) and MATLAB (version 23.2.0.2365128) was utilized. Images of the four samples scanned within a 20 μm × 20 μm area were selected and subjected to grayscale conversion, binarization, and other image processing steps.

**Figure 12:**
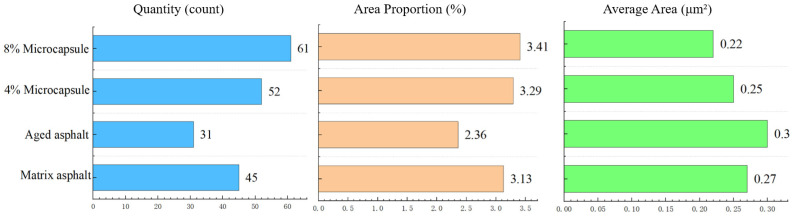
Characteristic parameters of bee-like structures in self-healing asphalt.

The results of parameters such as the number, area proportion, and average area of bee-like structures obtained from image analysis are shown in Figure 12.

(1) Characteristics of Bee-like Structures in Aged Asphalt

According to Figure 11, asphalt aging induces moderate evolution of the bee-like structures, which exhibit a more concentrated spatial distribution. Specifically, compared to the base asphalt, the average area of bee-like structures in aged asphalt increases by 11%, while their number and area proportion decrease by 31% and 25%, respectively. Aging leads to the formation of a film on the asphalt surface that inhibits the development of bee-like structures, increases the content of asphaltenes and resins in the asphalt, and results in a greater number of wax crystal nuclei [31]. These components merge and agglomerate on the surface of the wax crystal nuclei, thereby reducing both the number and area proportion of bee-like structures in aged asphalt.

For the microcapsule-modified self-healing asphalt investigated in this study, this aging-induced microstructural evolution is particularly critical. The surface oxidative film weakens the interfacial bonding between microcapsules and the asphalt matrix, increasing the risk of premature microcapsule debonding rather than triggered rupture under crack propagation. Furthermore, the rigid bee-like structures alter the crack propagation path, reducing the probability of cracks intersecting with microcapsules. The increased asphalt viscosity also hinders the diffusion and wetting of the released core healing agent on crack surfaces, ultimately compromising the long-term self-healing efficiency of the asphalt pavement. These findings provide essential microscale guidance for optimizing the anti-aging design of microcapsules and improving the durability of self-healing asphalt materials.

(2) Characteristics of Bee-like Structures in Self-Healing Asphalt

After incorporating 4% and 8% microcapsules, the bee-like structures in the self-healing asphalt show a certain degree of “shrinkage” compared to those in aged asphalt. The average area of the bee-like structures decreases by 17% and 27%, respectively, while both the number and area proportion of bee-like structures increase to varying degrees with the two microcapsule dosages. As the microcapsule content increases, the degree of “shrinkage” in the bee-like structures on the asphalt surface becomes more pronounced. This indicates that the corn oil rejuvenator increases the aromatic fraction content in aged asphalt, reduces the asphaltene content, and thereby slows down the trend of asphaltene merging and agglomeration, achieving regeneration of aged asphalt in the vicinity of cracks. This conclusion reveals, from a microscopic perspective, the mechanism by which microcapsules function within asphalt, demonstrating that microcapsule technology has a certain effect in enhancing the self-healing capacity of asphalt. This microstructural regeneration directly translates to improved self-healing capability. The smaller, more uniformly distributed bee-like structures reduce stress concentration points in the asphalt matrix, slowing down crack propagation. More importantly, the restored colloidal stability and increased molecular mobility of the asphalt matrix allow for better diffusion and wetting of the rejuvenator at crack interfaces, facilitating the re-bonding of cracked surfaces. This conclusion reveals, from a microscopic perspective, the mechanism by which microcapsules function within asphalt, demonstrating that microcapsule technology achieves targeted regeneration of aged asphalt in the vicinity of cracks and effectively enhances the long-term self-healing capacity of asphalt pavements.

(2) Analysis of Root Mean Square Roughness

The root mean square roughness ($R_{q}$) is a key parameter describing the surface roughness of a sample. It refers to the root mean square value of surface height and can accurately reflect the distribution of surface heights. Its definition is given in Equation (3).

(3) $$R_{q}=\sqrt{\frac{\iint {\left[\;y\left(a,b\right)-y_{0}\right]}^{2}\mathrm{ds}}{\iint \mathrm{ds}}}$$

where s—Size of the AFM scanning area (taken as 40 μm × 40 μm in this study); $y\left(a,b\right)$—Height function of the topography, in nm; $y_{0}$—Reference height, in nm.

**Figure 13:**
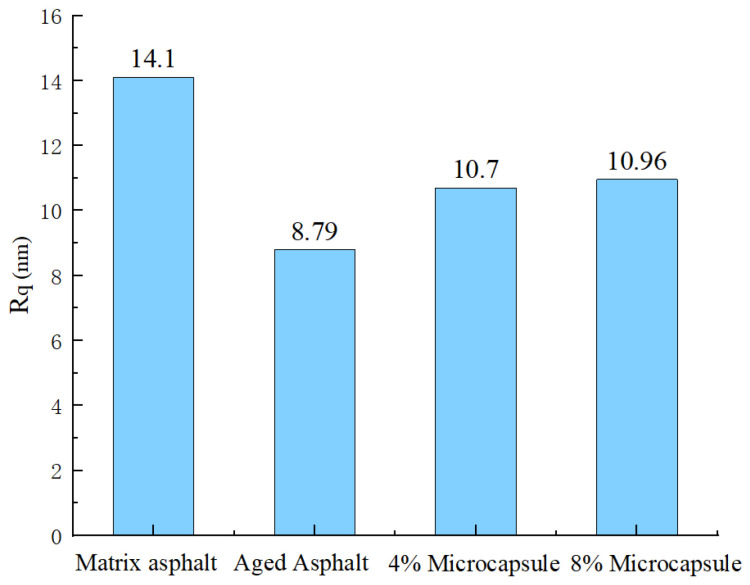
$R_{q}$ of each asphalt sample.

Allen et al. [32] utilized $R_{q}$ to investigate phase differences in asphalt at the microscopic scale, while Zhang et al. [33] found that $R_{q}$ can simultaneously quantify and analyze changes in “bee-like structures.” A larger $R_{q}$ indicates greater phase differences in asphalt at the microscopic level and more pronounced phase separation phenomena. To further quantitatively analyze the microscopic mechanism of how microcapsules function, the root mean square roughness $R_{q}$ was employed to quantify the influence of different microcapsule dosages on the microscopic surface morphology of asphalt. Using NanoScope Analysis software (version 1.40), the $R_{q}$ values of the four samples within a 40 μm × 40 μm scanning area were calculated, and the results are presented in Figure 13.

The $R_{q}$ value of the aged asphalt sample decreased by 5.31 nm compared to that of the base asphalt. This reduction is attributed to the migration and transformation of asphalt components during aging, which leads to an increase in the content of asphaltenes and resins. Consequently, the multiphase state of the asphalt tends to become more uniform [34], thereby reducing the differences in roughness.

In contrast, the $R_{q}$ values of the asphalt samples with 4% and 8% microcapsules decreased by only 3.4 nm and 3.14 nm, respectively, relative to the base asphalt. This indicates that the microcapsules mitigated the impact of asphalt aging on the $R_{q}$ value, achieving a certain degree of restoration of the aged asphalt, with repair rates of 36% and 41%, respectively. The addition and effective release of the microcapsules reduced the agglomeration of asphaltenes, promoted the accumulation and continuous growth of wax crystals, and slowed down the influence of aging on the bee-like structures. Moreover, this mitigating effect increased with higher microcapsule dosage. The corn oil rejuvenator released from the microcapsules reversed the aforementioned homogenization process, further validating the repair-promoting capability of the microcapsules on aged asphalt. From a functional perspective, maintaining appropriate surface roughness has significant engineering implications: it enhances asphalt-aggregate interfacial adhesion, improves pavement skid resistance, provides more crack healing nucleation sites, and facilitates rejuvenator diffusion and wetting on crack surfaces for better molecular interdiffusion and bonding, thus boosting asphalt self-healing efficiency. These results establish a clear quantitative correlation between microcapsule dosage, surface roughness evolution and microstructural repair effect, demonstrating that microcapsule technology not only repairs macroscopic cracks but also preserves asphalt’s nanoscale and microscale morphological integrity during aging, which is critical for long-term pavement performance and durability.

### 3.3. Macroscopic Self-Healing Behavior of Asphalt Mixture with Corn Oil Microcapsules

#### 3.3.1. Influence of Healing Temperature on Splitting Performance

**Figure 14:**
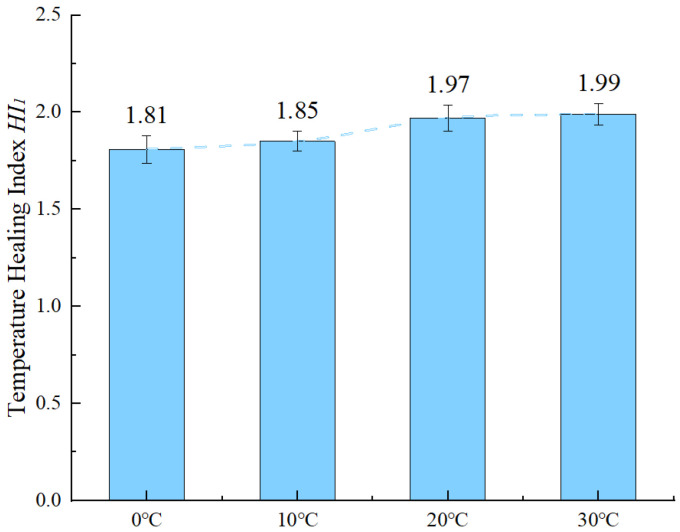
Healing index of self-healing asphalt at different healing temperatures.

In this study, Marshall specimens of microcapsule-modified asphalt concrete with a corn oil microcapsule content of 6% were used for splitting tests. During the two-stage splitting test described above, the curing temperatures were set at 0 °C, 10 °C, 20 °C, and 30 °C, respectively, with the curing time fixed at 4 h. The test results are presented in Figure 14.

From the results, it can be observed that as the environmental chamber temperature increased from 0 °C to 30 °C, the healing index of the Marshall specimens for the corn oil microcapsule-modified asphalt mixture rose from 1.81 to 1.99. This improvement is attributed to the gradual enhancement of asphalt flow performance with increasing healing temperature, which promotes the rearrangement and reorganization of asphalt molecules, thereby increasing the self-healing index.

These findings indicate that the self-healing effect of the Marshall specimens for the corn oil microcapsule-modified asphalt mixture significantly improves with an increase in healing temperature [35]. This suggests that higher temperatures enhance the healing capacity of the corn oil microcapsule-modified asphalt concrete. Furthermore, it implies that cracks generated under the influence of low winter temperatures can be restored when temperatures rise in summer. This behavior is likely due to the high temperature sensitivity of the Marshall specimens for the corn oil microcapsule-modified asphalt mixture, possibly because of their inherently low glass transition temperature, making the activity of asphalt molecules more susceptible to positive temperature gradients.

#### 3.3.2. Influence of Healing Time on Splitting Performance

**Figure 15:**
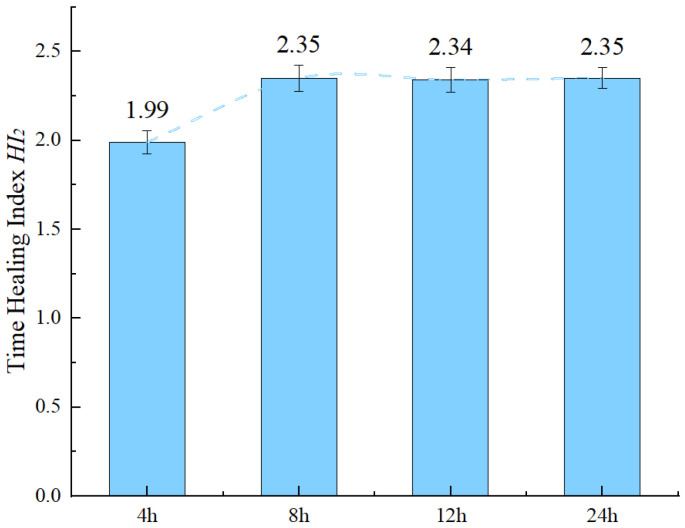
Healing index of self-healing asphalt at different healing times.

In the two-stage splitting test described above, the curing times were set to 4 h, 8 h, 12 h, and 24 h, respectively, with the curing temperature fixed at 30 °C. The healing time refers to the period allowed for the material to undergo its self-healing process after damage occurs due to external loading. The test results are presented in Figure 15.

From the results, it can be observed that when the healing time was extended from 4 h to 8 h, the healing index ${\mathrm{HI}}_{2}$ of the Marshall specimens for the corn oil microcapsule-modified asphalt mixture increased from 1.99 to 2.35. This indicates that the microcracks generated during the first stage of damage triggered the release of corn oil from the microcapsules. Once both sides of the crack were wetted, sufficient time was required for the small asphalt molecules to diffuse and penetrate the asphalt matrix, and the increase in fracture strength followed the principle of time superposition.

However, when the healing time was further extended beyond 8 h, the healing index did not show a significant further improvement. This is attributed to the limited content of corn oil microcapsules in each Marshall specimen. Even with additional healing time, no further enhancement in the self-healing performance of the corn oil microcapsule-modified asphalt concrete was observed.

#### 3.3.3. Influence of Corn Oil Microcapsule Content on Splitting Performance

In this study, to investigate the optimal dosage, Marshall specimens of microcapsule-modified asphalt concrete with corn oil microcapsule contents of 0%, 3%, 6%, 9%, and 12% were prepared for splitting tests. In the two-stage splitting test described above, the curing time was set to 8 h, and the curing temperature was fixed at 30 °C. The test results were shown in Table 4.

Analysis of the variation pattern of the healing index as the corn oil microcapsule content increased from 0% to 6% reveals that the self-healing effect of the Marshall specimens for the asphalt mixture gradually strengthened with the increase in microcapsule content. This indicates that the addition of corn oil microcapsules positively influences the healing performance of the asphalt mixture Marshall specimens. However, when the corn oil microcapsule content continued to increase, the healing index $HI_{3}$ did not increase but instead decreased. This suggests that there may be a peak in $HI_{3}$ between microcapsule contents of 6% and 12%. The underlying reason for this phenomenon is likely that an excessive amount of corn oil microcapsules can adversely affect the mechanical properties of the asphalt mixture to some extent, consequently leading to a decrease in the healing index.

**Figure 16:**
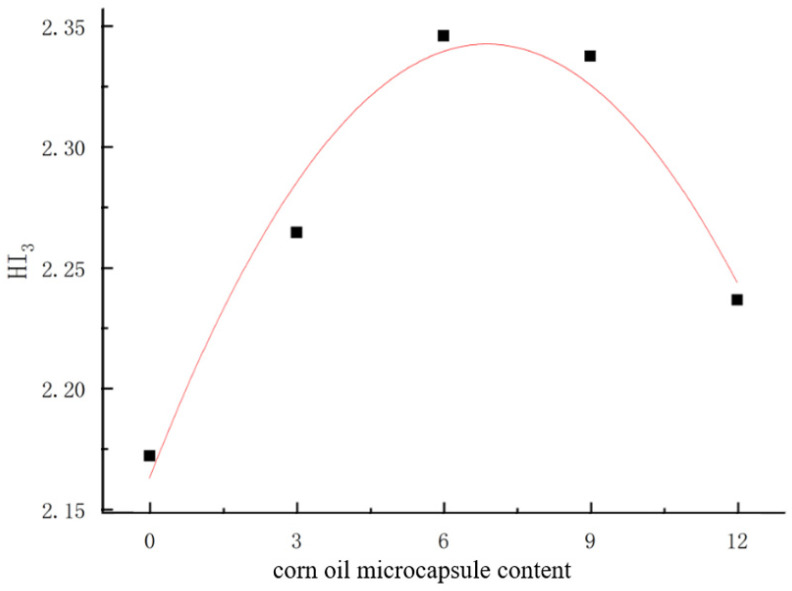
Correlation between corn oil microcapsule content and healing index of self-healing asphalt.

To determine the optimal content of corn oil microcapsules [36], a fitting analysis was performed on the test results mentioned above, as shown in Figure 16. The analysis shows that the correlation coefficient R^2^ between the corn oil microcapsule content and the healing index HI_3_ is 0.9646, indicating a strong correlation. The fitted formula is as follows:

(4) $$y=2.1631+0.0521x-0.0038x^{2}$$

where y is the healing index $HI_{3}$ (dimensionless); x is the corn oil microcapsule content (%).

Based on the equation, the healing index $HI_{3}$ reaches its maximum value when the corn oil microcapsule content is 6.86%. However, as the content increases from 6% to 6.86%, $HI_{3}$ does not show a significant change. In practical applications, to balance operational feasibility and economic efficiency, the microcapsule content should be rounded to the nearest whole number. Therefore, based on the healing index results, the recommended content of corn oil microcapsules within the tested range is 6%.

## 4. Conclusions

This study prepared microcapsules using corn oil as an asphalt rejuvenator and verified their fundamental properties, including high temperature resistance, salt resistance, and spectral characteristics. The microscopic surface features of the asphalt containing microcapsules were analyzed via atomic force microscopy. Microcapsule-modified asphalt mixture specimens were fabricated, and a splitting test was designed to evaluate their healing performance. The main conclusions are as follows:

(1) Using corn oil as the core material and a thermosetting resin (MUF) formed by the condensation of melamine, urea, and formaldehyde as the shell material, under the conditions of urea accounting for 20% of the prepolymer mass, a molar ratio of formaldehyde to melamine and urea of 2.5, and an emulsification rate of 1.2 kr/min, the resulting corn oil microcapsules exhibited high yield and uniform particle size. With increasing corn oil microcapsule content, the self-healing effect of the microcapsule-modified asphalt mixture gradually improved. The recommended content of corn oil microcapsules within the tested range is 6%.
(2) For asphalt samples containing 4% and 8% microcapsules, the average area of the bee-like structures on the surface decreased by 17% and 27%, respectively, and the recovery rates of the $R_{q}$ value were 36% and 41%, respectively. The addition of corn oil microcapsules reduced the impact of aging on the microscopic parameters of the asphalt surface, thereby partially restoring the aged asphalt.
(3) The self-healing performance of the corn oil microcapsule-modified asphalt mixture improved with increasing healing temperature. When the healing temperature rose from 0 °C to 30 °C, the healing index of the mixture increased from 1.81 to 1.99.
(4) Prolonging the healing time significantly enhanced the self-healing effect of the corn oil microcapsule-modified asphalt mixture. When the healing time was extended from 4 h to 8 h, the healing index ${\mathrm{HI}}_{2}$ increased from 1.99 to 2.35.

## Figures and Tables

**Table 1:** Sample codes with different urea mass ratios.

No.	1	2	3	4	5
urea mass ratio	0%	10%	20%	30%	80%
M_F_:M_(M+U)_	3.0	2.7	2.5	2.3	1.6

**Table 2:** Experimental results of prepolymer synthesis.

No.	1	2	3	4	5
Urea mass ratio	0%	10%	20%	30%	80%
M_F_:M_(M+U)_	3.0	2.7	2.5	2.3	1.6
Reaction time/min	14	23	77	82	96

**Table 3:** Salt tolerance test results.

Sample	$m_{1}$	$m_{2}$			$\mathrm{Average}\;m_{2}$	SD	Y
		**1**	**2**	**3**			
0%	1.95	1.87	1.91	1.89	1.89	0.02	3%
5%	1.93	1.65	1.64	1.66	1.65	0.01	14.5%
10%	1.95	1.57	1.55	1.59	1.57	0.02	19.5%

**Table 4:** Healing index of self-healing asphalt with different corn oil microcapsule contents.

Microcapsule Content/%	0	3	6	9	12
Healing Index/${\mathrm{HI}}_{3}$	2.17	2.26	2.35	2.34	2.24
SD	0.081	0.075	0.059	0.063	0.067

## Data Availability

The original contributions presented in this study are included in the article. Further inquiries can be directed to the corresponding author.
